# Assessment of the Oxidative Desulfurization of Middle Distillate Surrogate Fuels with Spectroscopic Techniques

**DOI:** 10.1155/2020/8876082

**Published:** 2020-12-09

**Authors:** Eleni Syntyhaki, Anastasia Detsi, Dimitrios Karonis

**Affiliations:** ^1^Laboratory of Fuels Technology and Lubricants, School of Chemical Engineering, National Technical University of Athens, Athens, Greece; ^2^Department of Chemistry, Fuels and Lubricants, Hellenic Navy General Staff, Athens, Greece; ^3^Laboratory of Organic Chemistry, School of Chemical Engineering, National Technical University of Athens, Athens, Greece

## Abstract

The oxidative desulfurization of five (5) model sulfur compounds and eleven (11) surrogate blends was investigated using the hydrogen peroxide (H_2_O_2_)-acetic acid (CH_3_COOH) system. Consequently, extractive desulfurization was carried out using conventional solvents. The model sulfur compounds, as well as the solvent, are present in petroleum middle distillates. The selection of the compounds was made so that they represent various kinds of sulfur compounds. The goal of this study was the implementation of a simple and economical oxidative and extractive system for the desulfurization of surrogate mixtures with an intermediate sulfur concentration 1% w/w, at the mild temperature of 70°C, and without the use of supplementary and assisting methods such as heterogeneous catalysis or ultrasound irradiation. The sulfur content was estimated using X-ray fluorescence. The progress of the oxidation reaction was monitored using liquid FT-IR. The solid sediments of the oxidation procedure were identified with solid-state FT-IR and ^1^H NMR spectroscopy.

## 1. Introduction

The strict international environmental regulation of the sulfur content in diesel fuels contributed substantially to the fact that deep desulfurization of middle distillates is a key issue in petroleum refineries. The conventional desulfurization method in industry is catalytic hydrodesulfurization (HDS). HDS process requires high selectivity catalysts and high consumption of hydrogen and operates at high temperature and pressure, leading to a large investment and operating cost. The types of sulfur compounds and the composition of the distillate affect the efficiency of the method. Benzothiophenes and dibenzothiophenes are highly resistant to hydrogenation, demanding more severe desulfurization conditions, due to their steric hindrance [1]. New alternative methods [2, 3] have been developed, in order to remove the refractory sulfur-containing compounds efficiently from the petroleum fractions, such as selective extraction [4], adsorption [5], oxidation [6], and biodesulfurization [7, 8].

Oxidative desulfurization (ODS) is currently receiving increasing attention and seems to be particularly promising since it avoids the use of hydrogen and can be conducted at mild conditions leading to a low operating cost. Moreover, ODS results in higher selectivity and efficiency. The refractory sulfur-containing compounds show high reactivity during the ODS process. The organosulfur compounds are oxidized to their corresponding sulfoxides or sulfones. Subsequently, these polarized products can be removed by extraction, adsorption, or filtration. Heterogeneous catalysis is an important assisting method in the ODS process. Transition metals such as molybdenum, vanadium, and tungsten have been reported as active phases, while various supports have been considered, including alumina, silica, and zeolites [9–11]. The use of ultrasound irradiation [12–14] was also investigated as an assisting technology in the ODS process. The introduction of ionic liquids and their distinctive properties induced their usage as cocatalysts in oxidative desulfurization, as well as solvents for extraction [15, 16].

ODS is carried out in the presence of a catalyst and an oxidant agent. Among the different oxidation systems that have been used, the hydrogen peroxide-carboxylic acid system [17–20], which produces in situ peracids, has major advantages, such as high-quality products, safety, low cost, and low environmental impact. In order to overpass the low mass transfer due to the low solubility of H_2_O_2_, cumene hydroperoxide [21] and cyclohexanone peroxide (CYHPO) [22] have also been proposed for ODS. Being oil-soluble oxidants, they have been found efficient at low sulfur feedstocks.

The ODS mechanism was introduced in 1966 by Heimlich and Wallace [23], according to the kinetic data acquired at 50–100°C, during the oxidation of white oil solutions of DBT by aqueous hydrogen peroxide-acetic acid solutions. The rate-determining step appeared to be the attack of a peracetic acid-hydrogen peroxide dimer on the sulfur atom of DBT. Zannikos et al. [24] studied the desulfurization of two straight-run gas oils with sulfur mass contents of 0.87% and 2.4% w/w using hydrogen peroxide and acetic acid as an oxidative system. The oxidation process itself led to the removal of a substantial portion of the sulfur that was originally present, without any negative effects on the other properties of the fuel. Furthermore, the extraction with removable solvents, such as methanol, led to overall desulfurization in excess of 90% at acceptable yields. That work introduced the possibility of desulfurizing the middle distillates of petroleum without recourse to hydrogen or the assistance of heterogeneous catalysis. A lot of efforts have been made using the hydrogen peroxide-carboxylic acid oxidative system in different blends, followed by extraction or absorption processes [25–29], in order to study the effects of the operating parameters. The oxidative desulfurization of four petroleum fractions with sulfur content 0.206–1.272% w/w was carried out at 50, 70, and 90°C, using the hydrogen peroxide-acetic acid system. ODS was followed by extraction with acetonitrile and methanol, resulting in high desulfurization yields for kerosene, light gas oil, and heavy gas oil [30]. The same oxidative and extractive system was studied in surrogate fuels with sulfur content 1%, at 50 and 70°C. The method was applied to eight different sulfur compounds and was significantly effective at 70°C reaction temperature [31].

Taking into consideration the abovementioned, the present study focuses on surrogate blends of sulfur concentration 1% w/w, which corresponds to a high sulfur gas oil. Both the selected model sulfur compounds and the hydrocarbon are representative of those contained in petroleum middle distillates. The efficiency of the hydrogen peroxide-acetic acid oxidative system was investigated under mild reaction temperature (70°C), in single, binary, ternary, and containing all five model sulfur compounds, model mixtures. Subsequently, extractive desulfurization was performed using common and affordable solvents.

Our goal was to implement a simple, flexible, economically affordable, and functional process at mild temperatures, without the use of assisting methods (heterogeneous catalysts or ultrasound irradiation) and with low-energy consumption.

Moreover, the detailed analysis results of the sulfur precursor compounds and their oxidized products included in this study, using liquid and solid FT-IR spectroscopic and ^1^H NMR techniques, are significant in petrochemical reactions.

## 2. Experimental

### 2.1. Materials

Sulfur compounds typical of those present in middle distillates were used. These compounds were 1-dodecanethiol representing mercaptans, n-butyl sulfide representing sulfides, tert-butyl disulfide representing disulfides, benzothiophene as benzothiophene, and dibenzothiophene. 1-Dodecanethiol > 95% was purchased from TCI, benzothiophene (BT) 98% was obtained from Flurochem, and n-butyl sulfide 99%, tert-butyl disulfide 98%, and dibenzothiophene (DBT) 98% were supplied from Acros Organics. N-hexadecane 95% was purchased from Alfa Aesar. Acetic acid 99% was obtained from Fluka. Hydrogen peroxide 30% w/v was purchased from PSR Panreac.

Acetonitrile HPLC Gradient Grade was obtained from Carlo Erba. Methanol 99.8% was supplied from Sigma-Aldrich. All of the reagents, hydrocarbon and solvents, were used as they were received.

### 2.2. Procedure

#### 2.2.1. Oxidation Procedure

In our study, the reaction mixtures were prepared using five model sulfur compounds that can be found in petroleum middle distillates. The selection of the compounds was made so that they represent various kinds of sulfur compounds such as thiols, sulfides, disulfides, and sulfur-containing aromatic compounds. In addition, they should be commercially available and economically feasible to purchase. The reaction solvent was n-hexadecane which is also present in petroleum middle distillates. The choice of a single solvent was made so that the model surrogate system would be as simple as possible.

It is known that the oxidative desulfurization of fuels with a sulfur content of 0.87–2.40% w/w, using the same oxidative system at 90°C reaction temperature, is particularly effective. Specifically, the desulfurization yield reaches 90% [24].

In our study, we chose the sulfur content in all of the mixtures, to have an intermediate value of 1% w/w, and also the oxidation temperature to be 70°C, since our intention was the reaction conditions to be milder. Moreover, a lot of preliminary tests were performed using the same substances at different reaction temperatures, in order to result in the specific oxidative scheme [31].

The content of each sulfur compound was determined so that its sulfur content was equally proportional to the final sulfur content of the reaction mixture. Specifically, in binary mixtures, the sulfur content of each sulfur compound was almost 0.5% w/w while in ternary mixtures the sulfur content of each sulfur compound was approximately 0.333% w/w. Then, in the mixture which contained all of the five sulfur compounds, the sulfur content of each one of them was about 0.2% w/w. The types of the sulfur compounds contained in the eleven different mixtures are depicted in Table 1.

Initially, the five sulfur compounds were individually oxidized in order to find out how they behave under the specific experimental conditions, and then the oxidation of their eleven mixtures was carried out.

The oxidation procedure took place in a boiling flat bottom flask equipped with a condenser, a magnetic stirrer, and a thermometer. During the reaction, the mixture was heated using a ceramic heater.

In each experimental run, the reaction temperature was about 70°C while the reaction time was set at 3 hours. The oxidations were carried out using 100 ml of the untreated mixtures and the required amount of the acetic acid, so that the molar ratio *n*_CH3COOH_*/n*_*S*_ was equal to 17. The mixture was inducted to the apparatus stirred continuously at 400 rpm constant mixing speed and heated to the desired reaction temperature of 70°C. When the system reached the selected reaction temperature, the aqueous solution 30% w/v of the hydrogen peroxide was added to the reaction mixture gradually in three steps. In each step, the system was allowed to settle and reach the standard temperature. The required amount of oxidant was estimated so that the molar ratio *n*_H2O2_*/n*_*S*_ was equal to 3. Since the mixture is a heterogeneous system of two phases, intense mixing was necessary to ensure the homogeneous composition of the bulk liquids. During that time, liquid samples were withdrawn from the reaction mixture every 30 minutes, in order to monitor the progress of the oxidation procedure using FT-IR. Finally, the system was allowed to settle and cool at room temperature, whereupon the two layers invariably formed. In six specific mixtures of the total eleven, a white solid precipitate was formed and separated from the liquid supernatant mixture. The white precipitate was filtered under vacuum, washed with deionized water at 100°C, and then dried in a desiccator under vacuum. Subsequently, the remaining liquid supernatant reaction mixture followed the standard extraction process of the rest of the mixtures. The oxidative desulfurization procedure, the isolation of any derived precipitate, and the separation and purification of the oxidized organic phase were repeated in order to confirm our results.

#### 2.2.2. Extraction Procedure

The reaction mixture was inducted into a 250 ml separation funnel to settle. Then, the organic phase and the aqueous phase were separated. In order to remove the traces of the acetic acid from the organic phase, the organic phase was washed with deionized water at 100°C. Subsequently, appropriate amounts of the washed organic phase and solvent (acetonitrile or methanol) were mixed according to the feed-to-solvent ratio of the experimental design (1 : 1, 1 : 1.5, 1 : 2). The mixtures were vigorously shaken and if necessary centrifuged. Then, the mixtures were transferred to small separation funnels to settle overnight. The n-hexadecane phases were separated from the solvent phases and their weights were measured. Finally, the extracted products were analyzed for their sulfur content, and their liquid FT-IR spectra were obtained. Both the extraction and the spectra acquisition were repeated in order to come to a safe conclusion.

### 2.3. Analysis

The FT-IR spectra of the liquid samples were acquired using IRAffinity-1 Fourier Transform Infrared Spectrometer (Shimadzu), equipped with a horizontal attenuated total reflectance sample cell made from zinc selenide (ZnSe) crystal.

The FT-IR spectra of the solid samples were recorded on the Jasco FT-IR-4200 spectrometer equipped with a TGR detector with an accumulation of 32 and a resolution of 4.0 cm^−1^. Dried KBr was used for the formation of the sample pellets.

The NMR spectra of the solid samples were recorded on Varian V600 MHz in the National Hellenic Research Foundation. The ^1^H NMR chemical shifts were referenced to the peak at 7.26 ppm of the dilution solvent CDCl_3_. In specific cases, (CD_3_)_2_SO was used; then, the ^1^H NMR chemical shifts were referenced to the peak at 2.50 ppm [32].

The sulfur content (% w/w) of the liquid samples was measured by X-ray fluorescence according to the ISO 8754 method using a SpectroXepos (Ametek) unit. Test portions from the samples were placed in the sample cups providing a sample depth of at least 3-4 mm. The sample cups are removable and equipped with replaceable X-ray transparent film 6 mm, made of polyester, polypropylene, or polycarbonate. The apparatus consists of a source of X-ray excitation with significant X-ray flux at energies above 2.5 keV, an X-ray detector with high sensitivity at 2.3 keV, and filters of discriminating between sulfur *Kα* radiation and other X-rays. The display provides a readout in sulfur content as a percentage by mass [% (m/m)].

## 3. Results and Discussion

### 3.1. Oxidation and Extraction of the Model Sulfur Compounds

The oxidation of the sulfur compounds increases their polarity, therefore, enhances their susceptibility, and hence, improves the yield of the desulfurized product. The acetic acid that was used in the oxidation is itself an extraction solvent for the oxidized sulfur compounds. The oxidation of benzothiophene and dibenzothiophene resulted in the precipitation of white solid residues, which were identified using the FT-IR and ^1^H NMR techniques.

As can be seen from Table 2, the oxidation of n-butyl sulfide, tert-butyl disulfide, benzothiophene, and dibenzothiophene resulted in satisfactory desulfurization yields to the extent of approximately 78–100%. Acetic acid seemed to be a quite effective solvent for removing the oxidized sulfur compounds. An exception is 1-dodecanethiol, where the desulfurization yield reached only 66%. Both acetonitrile and methanol proved to be efficient enough in the removal of oxidized sulfur compounds. Their efficiency increased with the rise of the solvent to feed (S/F) ratio. When the S/F ratio was equal to 2 and the extraction solvent was acetonitrile, the reduction of the sulfur content was 73% to 100%, whereas when methanol was the extraction solvent, the decrease of the sulfur content was 71% to 100%.

In general, the oxidation of pure sulfur compounds such as thiols, sulfides, and disulfides can easily lead to sulfonic acids [33]. In the case of aliphatic and aromatic thiols and sulfides, the sulfur oxidation path could be more complex due to the concurrent oxygen transfer from sulfoxide obtained as an intermediate oxidation product of the original sulfur compound [34, 35].

Thiols readily undergo oxidative coupling to give disulfides. Both radical and electrophile-nucleophile interactions may be involved. More powerful oxidizing agents convert thiols directly into sulfonic acids. The oxidation of thiols with peroxidic compounds is described by a typical reaction path where the initially formed product could be the corresponding disulfide. In particular, when hydrogen peroxide is used, it requires very long reaction times and proceeds only in strong acidic or basic conditions [33, 36].

The low desulfurization efficiency of 1-dodecanethiol, as outlined in Table 2, could be attributed to the low electron density of the sulfur atom. The only electron donor moiety, in this case, is the large alkyl group, consisting of twelve carbon atoms, and the effect of the inductive phenomenon (+*I*) is attenuated to the third carbon atom. Additionally, the solubility of thiols seems to affect the reaction rate: the longer the alkyl chain, the more difficult the oxidation. The oxidation reaction rates are also affected by solvents [33]. Other possible reasons could be, as aforementioned, the more complex oxidation path of thiols [19, 36] and the very long reaction times needed [33, 36].

Sulfides react with sources of electrophilic oxygen, e.g., peroxy acids. The first oxidation step, giving the sulfoxide, is often considerably faster than the second, which gives the sulfone and many sulfoxides may thus be prepared by this route. Vigorous reaction conditions lead directly to sulfones [37]. According to another point of view [35], indeed sulfoxides occur as intermediates; nevertheless, the rates of their formation compared with the rates of sulfones synthesis are faster with electrophilic oxidation agents such as peroxy acids and slower with nucleophilic oxidation agents such as peroxy anions [35].

The high desulfurization yield of n-butyl sulfide can be explained, since two n-butyl alkyl groups contribute to the sulfur atom electron density increase via a + *I* inductive effect, thus enhancing its nucleophilic character.

Oxidation of disulfides leads ultimately to sulfonic acids, but several intermediates (or their derivatives) frequently can be obtained such as thiosulfinate, thiolsulfonate, sulfinyl sulfone, and *α*-disulfone. Allen and Brook [38] were able to control oxidation of a symmetrical acyclic disulfide with 30% H_2_O_2_. Apparently, the sulfur-sulfur bond is more stable when the attached groups are aliphatic than when they are aromatic [33, 36, 38]. Peracids and hydrogen peroxide in acetic acid are the most common reagents used to oxidize disulfides. The large difference in the low reactivity of organic disulfides with both alkyl and aryl substituents combined with the relative sulfides can be ascribed to the substitution of an SR group for an *R* group. That may lower the nucleophilicity of the sulfur atom to which it is bound in the disulfide, thus lowering the rate by which it attacks the oxidizing reagent [39].

Considering the desulfurization capability of the tert-butyl disulfide, the three methyl groups of the tertiary butyl group amplify the electron density of the sulfur atoms due to the +*I* inductive effect. On the other hand, the S-tert-butyl group is electron-withdrawing (−*I*) and may lower the nucleophilicity of the sulfur atom to which it is bound, in the disulfide. Thus, the desulfurization ability of the tert-butyl disulfide is a combination of two phenomena, resulting in a desulfurization yield, which is higher than that of the 1-dodecanethiol and lower than that of the n-butyl sulfide.

Otsuki et al. [18] investigated the relationship between the electron densities of sulfur atoms in model sulfur compounds and their reactivities during the oxidation with hydrogen peroxide and formic acid. The electron density of benzothiophene (BT) was 5.739 and the electron density of dibenzothiophene (DBT) was 5.758. Model compounds with higher electron densities than BT were oxidized to form corresponding sulfones. When the electron density on the sulfur atom was higher, the rate constant of oxidation was higher. Thus, the apparent rate constants decreased in the order DBT > BT [4]. De Fillipis and Scarsella [19], using the same oxidative system, confirmed and extended the above literature data, pointing out the more complex oxidation path of thiols and sulfides, as highlighted by kinetic evidence, when compared with heterocyclic sulfur compounds such as benzo- and dibenzothiophene. Specifically, for benzothiophene, dibenzothiophene, and diphenyl sulfide, the corresponding sulfones are formed and are present as a solid residue, both in the organic and in the aqueous phases of the mixture. The oxidation kinetics of benzothiophene and dibenzothiophene was studied later [40] with the same oxidative system at 40°C indicating that the parallel oxidation reactions in the organic phase are actually two-step reactions. The first step, the respective sulfoxide formation, is the controlling step while the second step, the respective sulfone formation, is being characterized by a very high reaction rate.

In our study, the desulfurization yields of the aromatic sulfur compound mixtures are particularly high. That is something predictable, since in both cases white solid residues are separated, removed, and precipitate from the supernatant. Moreover, the higher desulfurization rate of the dibenzothiophene solution than the one of the benzothiophene solution can be justified from the aforementioned, which suggests its easier oxidation.

#### 3.1.1. Liquid-Phase FT-IR Results

The oxidative and extractive desulfurization results that are depicted in Table 2 can also be justified by the observation of the FT-IR spectra of the oxidation and extraction products.

The stretching vibrations assigned to the C-S linkage occur in the region of 700–600 cm^−1^, while the S-S stretching vibration falls between 500 and 400 cm^−1^. Both absorptions are very weak. These bands were out of the region of our spectra. The aliphatic mercaptans as liquids or in solution show S-H stretching absorption in the range of 2600–2550 cm^−1^. The stretching band is characteristically weak [41, 42] and could not be detected in our spectra.

Since n-hexadecane was the only solvent, its spectrum covered most of the peaks of the diluted sulfur compounds with a few exceptions.

The spectrum of 1-dodecanethiol solution was almost identical to the spectrum of n-hexadecane. In the spectrum of n-butyl sulfide solution, two additional peaks could be observed at 1273 and 1220 cm^−1^ due to the methyne skeletal C-C vibrations.

In general, the tertiary butyl group gives rise to two C-H bending bands, one in the 1395–1385 cm^−1^ region and one near 1370 cm^−1^. The long-wavelength band is more intense [41, 42]. In the case of the tert-butyl disulfide solution, only the more intense peak of this doublet could be observed at 1363 cm^−1^, since most of the spectrum was covered by n-hexadecane absorptions.

In the solutions of the sulfur-containing aromatic compounds, benzothiophene and dibenzothiophene which are the most prominent and informative strong absorption bands that result from the out-of-plane (“oop”) bending of the ring C-H bonds appeared in the low-frequency range between 900 and 700 cm^−1^. Low-intensity in-plane bending bands also appeared in the 1300–1000 cm^−1^.

Specifically, in the benzothiophene solution, the out-of-plane peaks appeared at 798, 760, and 734 cm^−1^, while several low-intensity “oop” and in-plane peaks were also observed. The FT-IR spectrum of the dibenzothiophene solution showed only one out-of-plane peak at 740 cm^−1^ and a number of low-intensity in-plane peaks.

The characteristic peaks of the FT-IR spectra of the initial sulfur compound solutions are summarized in Table 3. The absorptions of the solvent n-hexadecane were excluded.

Primarily, the sulfones' spectra show strong absorption bands at 1350–1300 and 1160–1120 cm^−1^. These bands arise from asymmetric and symmetric stretching SO_2_, respectively. Hydrogen bonding results in absorption near 1300 and 1125 cm^−1^. Alkyl and aryl sulfoxides show strong absorption in the 1070–1030 cm^−1^ region. Since the sulfoxide group is also susceptible to hydrogen bonding, the absorption shifts to slightly lower frequencies [41, 42].

The monitoring of the progress of the oxidation procedure was controlled at regular time intervals, by examining the FT-IR spectra of aliquots that were withdrawn from the reaction mixture. The acetic acid, present in the reaction mixture, showed an intense C=O stretching band at 1716 cm^−1^ and a band arising from C-O stretching near 1289 cm^−1^ [41, 42]. During the oxidation of the model sulfur compounds, the C-O stretching peak overlapped the intensity of the more distinguishable sulfone peak near 1300 cm^−1^. Nevertheless, the sulfone peak near 1160–1120 cm^−1^ and the sulfoxide peak near 1070–1030 cm^−1^ were used for the monitoring of the oxidative reaction.

Of particular interest is the study of the FT-IR spectra of 1-dodecanethiol oxidation, since 1-dodecanethiol exhibits the lowest desulfurization yield, after both the oxidation and the extraction of its oxidized product. The observation of the sulfone and sulfoxide peaks at 1133 and 1060 cm^−1^, respectively, showed that sulfones were formed from the first hour of the reaction procedure and remained in the oxidized product. On the contrary, the intensity of the sulfoxide peak took its maximum value in the first hour of the oxidation, decreased and disappeared during the reaction. The same peak corresponding to sulfones was also evident in the FT-IR spectra of the extracted products.

As aforementioned, thiols follow a more complex oxidation path which starts with the formation of disulfides. Additionally, the longer the alkyl chain, the more difficult the oxidation. Therefore, it could be assumed that the oxidation of 1-dodecanethiol leads to the formation of sulfones and other sulfur species. These oxidized species remain in the organic phase of the reaction mixture. Such oxidized products, due to their large alkyl chain, lose their bipolar character and therefore cannot be extracted effectively. Thus, initially acetic acid cannot function as an effective solvent removing them from the organic to aqueous phase, and thereafter acetonitrile and methanol cannot extract them efficiently from the oxidized phase.

The n-butyl sulfide solution showed a high desulfurization rate. In the FT-IR spectra obtained at specific time intervals during its oxidation procedure, a peak appeared at 1136 cm^−1^ which can be attributed to the formation of the polar sulfones. The intensity of the peak took its maximum value at the first hour of the oxidative reaction and then decreased, due to the removal of the sulfones from the organic to aqueous phase of the mixture. The FT-IR spectrum of the oxidized product was identical with the spectrum of n-hexadecane, suggesting the removal of polarized oxidized molecules.

Since the original FT-IR spectrum of the tert-butyl disulfide solution showed absorptions in the sulfone region, the respective sulfone peaks could not contribute to the observation of the gradual oxidation of the mixture. Nevertheless, the appearance of a sulfoxide peak at 1060 cm^−1^ was used for monitoring the oxidation. Even though sulfoxides were formed from the first hour of the reaction, the intensity of the sulfoxide peak was higher at the second to the third hour of the oxidation procedure. The FT-IR spectrum of the tert-butyl disulfide oxidized solution was similar to the one of n-hexadecane.

The oxidation of the benzothiophene solution resulted in the formation of a white solid precipitate. The FT-IR spectra of supernatant samples withdrawn at regular intervals from the reaction mixture showed that the initial peaks involving aromatic ring absorptions disappeared from the first half-hour of the oxidation reaction. The FT-IR spectra of the aliquots were similar to the n-hexadecane FT-IR spectrum, apart from the presence of the absorption bands due to acetic acid.

**Figure 1:**
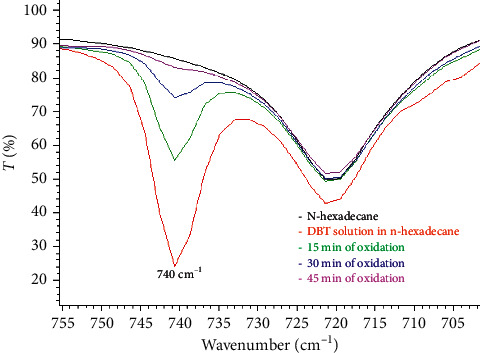
The intensity change of the characteristic “oop” peak of dibenzothiophene during 15, 30, and 45 minutes of oxidation.

The oxidation of the dibenzothiophene solution led also to the synthesis of a white solid precipitate. The FT-IR spectra of the reaction mixture aliquots showed that the out-of-plane bending of the ring C-H peak at 740 cm^−1^ was diminished over time as dibenzothiophene was oxidized, and its oxidized products were precipitated from the supernatant. Therefore, as depicted in Figure 1, the intensity of the “oop” peak gradually decreases and finally disappears from the first 45 minutes of the reaction. The FT-IR spectrum of the oxidized supernatant was similar to that of n-hexadecane.

The absorptions of the characteristic groups that contributed to the monitoring of the oxidation procedure in every sulfur compound solution are shown in Table 3.

In the FT-IR spectra of the extracted products with acetonitrile and methanol, a characteristic peak emerged at 1027–1029 cm^−1^, which possibly comes from the C-O stretching vibration of methanol, which remained in the phase of the extracted products.

The aforementioned spectroscopic data are in agreement with the desulfurization results.

#### 3.1.2. Solid-State FT-IR Results

**Figure 2:**
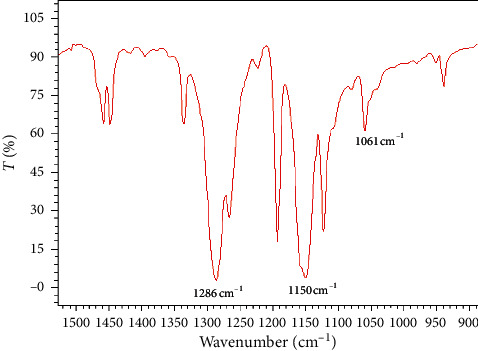
Solid-state FT-IR spectrum of the residual solid resulting from the benzothiophene oxidation.

The identification of the white solid resulting from the oxidation of benzothiophene showed that sulfone was formed since in its solid-state FT-IR spectrum two absorbance bands appeared at 1286 and 1150 cm^−1^, which are characteristics of the asymmetric and symmetric SO_2_ stretching. The absorption at 1061 cm^−1^ due to sulfoxides was minor. The solid-state FT-IR spectrum of the residual solid resulting from benzothiophene oxidation is shown in Figure 2.

**Figure 3:**
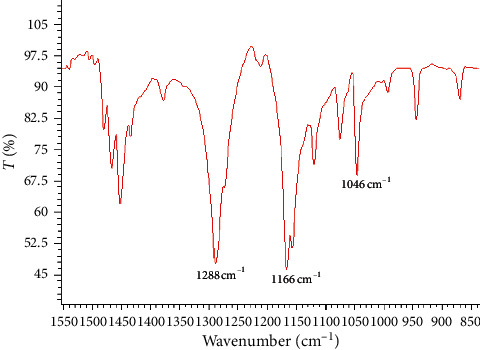
Solid-state FT-IR spectrum of the residual solid resulting from the dibenzothiophene oxidation.

Similarly, the characterization of the white residual substance produced from dibenzothiophene oxidation indicated that sulfone was synthesized because of the strong absorptions at 1288 and 1166 cm^−1^ [43] and the weak one at 1046 cm^−1^. The solid-state FT-IR spectrum of the residual solid resulting from dibenzothiophene oxidation is depicted in Figure 3.

#### 3.1.3. NMR Results

Initially, predictions of the NMR spectra of both sulfoxide and sulfone structures of benzothiophene and dibenzothiophene were made.

**Figure 4:**
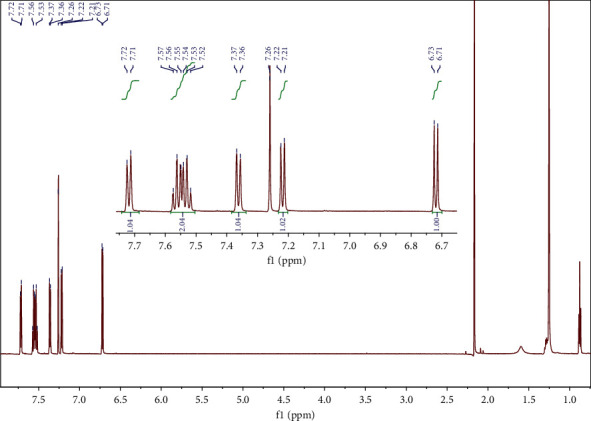
^1^H NMR of benzothiophene sulfone in CDCl_3_.

In the benzothiophene oxidized precipitate, the ^1^H NMR data are consistent with the bibliography [44] and confirm the results of the solid-state FT-IR according to which the oxidized product is the sulfone. Specifically and as illustrated in Figure 4, ^1^H NMR (CDCl_3_, 600 MHz): *δ* = 6.72 (d, *J* = 6.9 Hz, 1H), 7.22 (d, *J* = 7.3 Hz, 1H), 7.36 (d, *J* = 7.0 Hz, 1H), 7.55 (dt, *J* = 14.0, 6.6 Hz, 2H), and 7.72 (d, *J* = 7.4 Hz, 1H).

**Figure 5:**
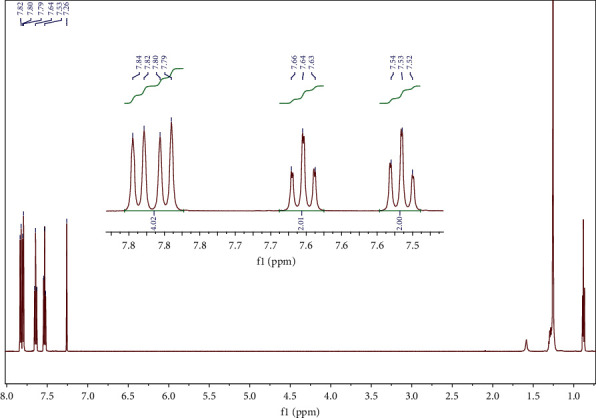
^1^H NMR of dibenzothiophene sulfone in CDCl_3_.

**Figure 6:**
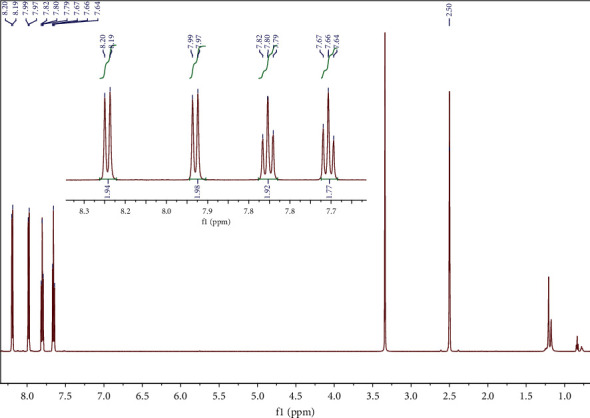
^1^H NMR of dibenzothiophene sulfone in (CD_3_)_2_SO.

The identification of the white solid precipitate resulting from the dibenzothiophene oxidation verified the conclusions obtained from the solid-state FT-IR spectrum that the residue compound is the sulfone. In fact, the NMR spectra were obtained in two different solvents CDCl_3_ and (CD_3_)_2_SO and in each case are consistent with the bibliography [45, 46] as depicted in Figures 5 and 6. Therefore, ^1^H NMR (CDCl_3_, 600 MHz): *δ* = 7.53 (t, *J* = 7.6 Hz, 2H), 7.64 (t, *J* = 7.6 Hz, 2H), 7.85–7.77 (m, 4H) and ^1^H NMR (CD_3_)_2_SO, 600 MHz): *δ* = 7.66 (t, *J* = 7.6 Hz, 2H), 7.80 (t, *J* = 7.6 Hz, 2H), 7.98 (d, *J* = 7.7 Hz, 2H), and 8.19 (d, *J* = 7.7 Hz, 2H).

### 3.2. Oxidation and Extraction of the Model Sulfur Compound Mixtures

Eleven model sulfur compound mixtures were made using n-hexadecane as solvent and five representative sulfur substances. Both solvent and sulfur compounds are present in the petroleum middle distillates. The scope was for the mixtures to be as simple as possible, in order to study the interaction of each sulfur compound in the final blend. For this purpose, initially, the oxidative desulfurization was carried out in binary systems, gradually in ternary systems adding a sulfur substance, which appeared to have quite interesting behavior, and finally in a blend which contained all five sulfur compounds in the same proportion of sulfur content. The composition of the eleven mixtures in the representative sulfur compounds is depicted in Table 1. In the mixtures 5, 6, 7, 8, 9, and 10, white solid residues were precipitated and identified by solid-state FT-IR and ^1^H NMR techniques. The results of the oxidative desulfurization and the extraction process of the eleven mixtures are presented in Table 4.

It is obvious that the five mixtures (6, 7, 8, 9, and 10) containing dibenzothiophene have quite high desulfurization yields ranging from 92 to 97%. The spectroscopic studies carried out in the sediments of the mixtures 6, 7, 9, and 10 showed that they consisted of dibenzothiophene sulfone, irrespective of the presence of benzothiophene in the mixture. The electron density of benzothiophene is higher than that of benzothiophene [18]; therefore, its reactivity is also higher [47]. Additionally, the benzothiophene-S-oxides are less stable than dibenzothiophene-S-oxides [48] resulting in the exclusive formation of dibenzothiophene sulfone under the specific experimental conditions.

Mixture 8 consisted of dibenzothiophene, benzothiophene, and n-butyl sulfide. In the sediment precipitated from mixture 8, both benzothiophene sulfone and dibenzothiophene sulfone exist at a ratio of about 1 to 3. This fact could be explained taking into account that n-butyl sulfide is easily oxidized, allowing the progressive oxidation of the small amount of benzothiophene to its sulfone form, in the ternary mixture. The extraction of the supernatants of the abovementioned mixtures had also high desulfurization rates. The efficiency of both acetonitrile and methanol increases with the raise of the solvent to feed (S/F) ratio. For S/F ratio equal to 2, the reduction of sulfur content is 94% to 100%.

Efficient desulfurization also takes place in binary mixtures, which contain n-butyl sulfide, which is more susceptible to oxidation than the aliphatic compounds 1-dodecanethiol and tert-butyl disulfide. The desulfurization rate reaches 92–98%. In the extraction procedure, when the S/F ratio is equal to 2, the reduction of sulfur content ranges from 94% to 99%.

The lowest rate of 73% desulfurization occurs in the binary mixture of 1-dodecanethiol and tert-butyl disulfide, and the decrease of sulfur content reaches 79% and 80% when the extraction solvent is acetonitrile and methanol, respectively. Therefore, it seems that the presence of n-butyl sulfide facilitates the oxidation of both 1-dodecanethiol and tert-butyl disulfide. This hypothesis can be consolidated taking into consideration the behavior of the ternary mixture 11, consisting of 1-dodecanethiol, n-butyl sulfide, and tert-butyl disulfide. Its desulfurization rate increases to 87% and the reduction of sulfur concentration after the extractions is 91–93%.

Of particular interest are the two binary mixtures 1 and 5, wherein one of the two substances is benzothiophene, and the other substances are tert-butyl disulfide and 1-dodecanethiol, respectively. These solutions have almost identical desulfurization rates 88% (mixture 1) and 89% (mixture 5). The sulfur reduction during the extractions is 95-96% with acetonitrile and 94-95% with methanol. Nevertheless, only in the case of mixture 5, a white precipitate is observed, which is identified as benzothiophene sulfone, according to the spectroscopic data set forth below. Consequently, it can be assumed that the difficulty in mercaptan oxidation, under the specific experimental conditions, facilitates the oxidation of benzothiophene to sulfone. It should be noted that the sulfoxide of benzothiophene cannot be isolated, as it is reported using various oxidants [47, 49].

Regarding the extraction process, both solvents are efficient for the removal of oxidized sulfur compounds, even though acetonitrile prevails. Their efficiency increases with increasing S/F ratio. Vice versa, the mass yields decrease without excessive changes, as the S/F ratio rises. In most cases, the mass yields are higher in methanol. Since the density of acetonitrile (0.786 g/cm^3^, 15°C) is closer than that of methanol (0.792 g/cm^3^, 15°C) to the density of n-hexadecane (0.770 g/cm^3^, 15°C), the mass loss is higher in the mixtures extracted with acetonitrile due to poor phase separation.

#### 3.2.1. Liquid-Phase FT-IR Results

The characteristic peaks of the FT-IR spectra of the initial surrogate fuels are presented in Table 5. The absorptions of the solvent n-hexadecane were excluded. Additionally, in Table 5 are depicted the absorptions of the characteristic groups that were used for the monitoring of the oxidation procedure.

In the liquid-phase FT-IR spectrum of mixture 1 (tert-butyl disulfide, benzothiophene), an absorption peak at 1161 cm^−1^ can be observed due to the skeletal C-C vibrations, and the out-of-plane (“oop”) peaks at 798, 760, and 734 cm^−1^ can be observed due to the aromatics C-H of benzothiophene. Most of the low-intensity “oop” and in-plane-peaks disappeared from the first 30 minutes of the oxidation, since the polar oxidized species moved from the organic to the aqueous phase of the biphasic mixture. Only the “oop” peak at 760 cm^−1^ remained, since benzothiophene was not completely oxidized as a solid sulfone precipitate. This absorption band diminished during the extraction procedure with acetonitrile and methanol.

The FT-IR spectrum of mixture 2 (1-dodecanethiol, tert-butyl disulfide) resembled the spectrum of n-hexadecane, since the latter prevailed. The only difference was the peak at 1161 cm^−1^. During the first 30 minutes of the oxidation process, the sulfone and sulfoxide peaks emerged at 1133 and 1060 cm^−1^, respectively, and remained in the oxidized product after the end of the reaction. These peaks insisted to be present in the product, even after the extraction process using acetonitrile and methanol. This behavior was also observed previously in the oxidation of the solution of the single 1-dodecanethiol. These spectroscopic observations are consistent with the experimental data, according to which the lowest rate of desulfurization 73% is present in the binary mixture of 1-dodecanethiol and tert-butyl disulfide.

The FT-IR spectrum of mixture 3 (1-dodecanethiol, n-butyl sulfide) is also similar to the one of n-hexadecane. The peak at 1135 cm^−1^ which can be assigned to sulfones appeared from the first half-hour of the reaction and took its maximum value of 1.5–2.0 hours of the oxidation procedure. The same pattern was observed for the peak attributed to sulfoxides at 1061 cm^−1^. Both peaks diminished during the extractions.

In the FT-IR spectrum of mixture 4 (n-butyl sulfide, tert-butyl disulfide), the characteristic peak of tert-butyl disulfide showed up at 1161 cm^−1^. As in the previous mixture, the sulfone peak at 1133 cm^−1^ was higher during 1.5–2.0 hours from the beginning of the oxidation reaction procedure, while it disappeared during the extractions, resulting in a spectrum similar to the one of n-hexadecane. The spectroscopic results of mixtures 3 and 4 are in accordance with their desulfurization yields which reach 92% and 98%, respectively.

The liquid spectrum FT-IR of mixture 5 (1-dodecanethiol, benzothiophene) is specifically important, due to the presence of benzothiophene in its oxidized form as a sulfone in the solid precipitate. In the spectrum of the initial mixture, the out-of-plane bending ring C-H peaks could be observed at 798, 760, and 734 cm^−1^. The low-intensity “oop” and in-plane- peaks disappeared from the first 30 minutes of the reaction. The peak at 760 cm^−1^, which diminished during the oxidation procedure, remained in the oxidized supernatant, since a quantity of benzothiophene was still present in its nonoxidized form and was eliminated from the spectrum during the extractions of the organic phase using acetonitrile and methanol.

The two binary mixtures 6 (1-dodecanethiol, dibenzothiophene) and 7 (tert-butyl disulfide, dibenzothiophene) had similar liquid FT-IR spectra and behavior. In both mixtures, white sediments precipitated, consisting only of dibenzothiophene sulfone. The spectra of the original nonoxidized samples showed an out-of-plane peak at 740 cm^−1^ and a number of low-intensity in-plane peaks. All of them were eliminated from the first 30 minutes of the oxidation in both mixtures. The FT-IR spectra of the extracted supernatant were identical to that of n-hexadecane.

**Figure 7:**
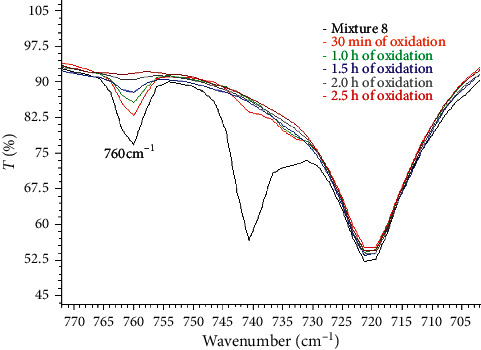
FT-IR liquid spectra of mixture 8. Peaks of benzothiophene and dibenzothiophene in the out-of-plane bending C-H region are presented.

Mixture 8 (n-butyl sulfide, benzothiophene, and dibenzothiophene) is of particular importance, since the sediment precipitated consisted of both sulfones of benzothiophene and dibenzothiophene. In the FT-IR spectrum of the primary mixture, the “oop” peaks attributed to benzothiophene and dibenzothiophene appeared at 760, 797, 866, and 741 cm^−1^, respectively, while several low-intensity in-plane peaks also emerged. All those peaks disappeared from the first 30 minutes of the oxidation procedure, apart from the main absorption peak of benzothiophene at 760 cm^−1^. As depicted in Figure 7, this peak diminishes during the reaction and finally disappears after 2.5 hours of reaction time. The FT-IR spectrum of the oxidized and extracted product was similar to the one of n-hexadecane. The gradual oxidation of benzothiophene is consistent with the formation and presence of its stable oxidized form of sulfone which exists in the precipitate of the ternary mixture.

In mixture 9, all sulfur compounds were present, aliphatic and aromatic. Since the concentration of every sulfur compound was low, n-hexadecane was prevalent. So, in the FT-IR spectrum of the initial mixture, the following peaks could be observed: the high intensity “oop” peaks of benzothiophene at 760 cm^−1^ and dibenzothiophene at 740 cm^−1^, as well as a number of low-intensity peaks both “oop” and in-plane presented in Table 5. These peaks were eliminated from the first half-hour of the reaction, and the spectrum of the oxidized and extracted supernatant was almost identical to the spectrum of n-hexadecane. A peak that appeared at 1133 cm^−1^ during the first 30 minutes of the oxidation could be attributed to sulfone formation.

The FT-IR spectrum and the behavior of mixture 10 (1-dodecanethiol, benzothiophene, and dibenzothiophene) were more or less similar to mixture 9. The only difference was that two low-intensity out-of-plane peaks at 848 and 866 cm^−1^ and one in-plane peak at 1056 cm^−1^ appeared in addition to the existing ones of mixture 9. All of those peaks disappeared from the first 30 minutes of the oxidation, and the FT-IR spectrum of the resulting product was very much like the one of n-hexadecane.

Mixture 11 consisted of aliphatic sulfur compounds (1-dodecanethiol, n-butyl sulfide, and tert-butyl disulfide). Its FT-IR spectrum resembled the one of n-hexadecane, since the latter prevailed. During the first half-hour of the oxidation procedure, the sulfone and sulfoxide peaks emerged at 1135 and 1061 cm^−1^, respectively, taking their maximum intensity values after 1.5 hours of reaction. The sulfone peak remained in the oxidized product and insisted to be present, even after the extraction process using acetonitrile and methanol. This behavior was mentioned above in the oxidation of the 1-dodecanethiol solution and mixture 2.

In some cases, during the extractions of the oxidized mixtures with methanol, an absorption appeared in the region of 1022–1028 cm^−1^, due to the C-O stretching vibration of the traces of methanol.

#### 3.2.2. Solid-State FT-IR Results

The results from the solid-state FT-IR spectra are analyzed hereinafter.

In the solid-state FT-IR spectrum of the precipitate from mixture 5 (1-dodecanethiol/benzothiophene), the characteristic sulfone bands were present at 1286 cm^−1^* ν*_*as*_(S=O) and 1135 cm^−1^* ν*_*s*_(S=O), but they were weak. Therefore, the accurate assessment of the sulfone formation was clarified from the ^1^H NMR spectrum of the precipitate.

The precipitates from mixture 6 (1-dodecanethiol/dibenzothiophene) and mixture 7 (tert-butyl disulfide/dibenzothiophene) are sulfones, since the characteristic sulfone bands were present and strong at 1288 cm^−1^* ν*_*as*_(S=O) and 1166 cm^−1^* ν*_*s*_(S=O).

The precipitate from mixture 8 (n-butyl sulfide/benzothiophene/dibenzothiophene) was also sulfone since the characteristic sulfone bands were evident and intense at 1287 cm^−1^* ν*_*as*_(S=O) and 1156 cm^−1^* ν*_*s*_(S=O).

Finally, the precipitates from mixture 9 (1-dodecanethiol/n-butyl sulfide/tert-butyl disulfide/benzothiophene/dibenzothiophene) and mixture 10 (1-dodecanethiol/benzothiophene/dibenzothiophene) were sulfones too, since the characteristic sulfone bands were present and strong at 1288 cm^−1^* ν*_*as*_(S=O) and 1166 cm^−1^* ν*_*s*_(S=O).

#### 3.2.3. NMR Results

The precipitates were dissolved in CDCl_3_. The NMR results confirmed the data obtained from the solid-state FT-IR spectra. Therefore, in each case, the dioxides of the corresponding aromatic sulfur compound were formed.

The precipitate from mixture 5 (1-dodecanethiol/benzothiophene) was benzothiophene sulfone ^1^H NMR (CDCl_3_, 600 MHz): *δ* = 6.72 (d, *J* = 6.9 Hz, 1H), 7.22 (d, *J* = 6.9 Hz, 1H), 7.36 (d, *J* = 7.0 Hz, 1H), 7.55 (dt, *J* = 15.0, 6.9 Hz, 2H), 7.72 (d, *J* = 7.3 Hz, 1H).

The precipitate from mixture 6 (1-dodecanethiol/dibenzothiophene) was dibenzothiophene sulfone ^1^H NMR (CDCl_3_, 600 MHz): *δ* = 7.53 (t, *J* = 7.2 Hz, 2H), 7.64 (t, *J* = 7.6 Hz, 2H), 7.84–7.78 (m, 4H).

The precipitate from mixture 7 (tert-butyl disulfide/dibenzothiophene) was dibenzothiophene sulfone ^1^H NMR (CDCl_3_, 600 MHz): *δ* = 7.52 (t, *J* = 7.6 Hz, 2H), 7.63 (t, *J* = 7.6 Hz, 2H), 7.84–7.76 (m, 4H).

**Figure 8:**
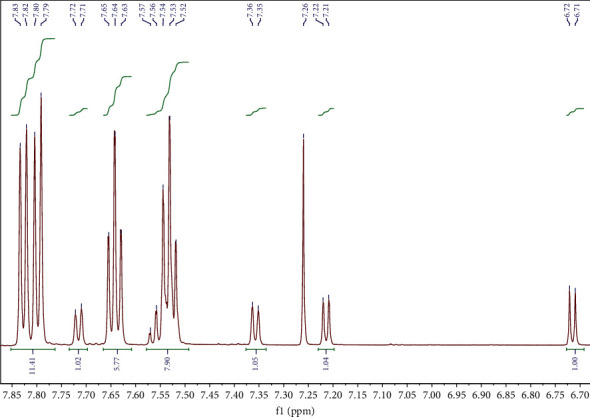
^1^H NMR of the precipitate from mixture 8 (n-butyl sulfide/benzothiophene/dibenzothiophene) in CDCl_3_ containing both dibenzothiophene sulfone and benzothiophene sulfone, at a ratio of approximately 3 : 1.

The precipitate from mixture 8 (n-butyl sulfide/benzothiophene/dibenzothiophene) consisted of both dibenzothiophene sulfone and benzothiophene sulfone at a ratio of approximately 3 : 1. Dibenzothiophene sulfone ^1^H NMR (CDCl_3_, 600 MHz): *δ* = 7.53 (t, *J* = 8.0 Hz, 2H), 7.64 (t, *J* = 7.6 Hz, 2H), 7.85–7.78 (m, 4H); benzothiophene sulfone ^1^H NMR (CDCl_3_, 600 MHz): *δ* = 6.72 (d, *J* = 6.9 Hz, 1H), 7.21 (d, *J* = 7.5 Hz, 1H), 7.36 (d, *J* = 6.9 Hz, 1H), 7.56–7.50 (m, 2H), 7.72 (d, *J* = 7.3 Hz, 1H). The ^1^H NMR spectrum of the precipitate from mixture 8 is shown in Figure 8.

The precipitate from mixture 9 (1-dodecanethiol/n-butyl sulfide/tert-butyl disulfide/benzothiophene/dibenzothiophene) was dibenzothiophene sulfone ^1^H NMR (CDCl_3_, 600 MHz): *δ* = 7.53 (t, *J* = 7.6 Hz, 2H), 7.64 (t, *J* = 7.6 Hz, 2H), 7.84–7.78 (m, 4H).

The precipitate from mixture 10 (1-dodecanethiol/benzothiophene/dibenzothiophene) was dibenzothiophene sulfone ^1^H NMR (CDCl_3_, 600 MHz): *δ* = 7.53 (t, *J* = 7.6 Hz, 2H), 7.64 (t, *J* = 7.6 Hz, 2H), 7.85–7.77 (m, 4H).

## 4. Conclusions

The purpose of this study was to implement a simple, mild, and economically affordable oxidative and extractive system, which is not supplemented or assisted by other methods, in the desulfurization of surrogate sulfur compound mixtures of 1% w/w. The progress of the reaction was monitored using liquid FT-IR. The identification of the solid sediments of the oxidation procedure was carried out, using solid-state FT-IR and ^1^H NMR spectroscopy. We came to the following conclusions:

1. The oxidative system of hydrogen peroxide and acetic acid at the mild temperature of 70°C is efficient for the completion of the oxidation reaction.
2. The low desulfurization capacity of 1-dodecanethiol may be due to its long alkyl chain, the more complex oxidation path of thiols, and the very long reaction times needed, when hydrogen peroxide is used.
3. The oxidation of the model aromatic sulfur compounds benzothiophene and dibenzothiophene under the specific conditions induced the oxidized formations of sulfones. In the more complex sulfur compound mixtures, the precipitate consisted mostly of dibenzothiophene sulfone, since dibenzothiophene sulfone predominates over benzothiophene sulfone.
4. Depending on the type/s of the sulfur compound/s, it was possible to observe the progress of the oxidation and when completed, focusing on the absorption bands of the aromatics' out-of-plane C-H bending and the stretching vibrations of sulfones and sulfoxides.
5. In every case, the data obtained from the liquid FT-IR spectra match and correspond to the properties and characteristics of the oxidized and extracted products.
6. Both extraction solvents acetonitrile and methanol proved to be efficient enough for the oxidized sulfur compounds removal, even though acetonitrile prevails. Their efficiency increases with the raise of the S/F ratio.

## Figures and Tables

**Table 1:** Model sulfur compounds contained in the eleven mixtures of surrogate fuels.

Mixture	Sulfur compounds
1	Tert-butyl disulfide, benzothiophene
2	1-dodecanethiol, tert-butyl disulfide
3	1-dodecanethiol, n-butyl sulfide
4	Ν-butyl sulfide, tert-butyl disulfide
5	1-dodecanethiol, benzothiophene
6	1-dodecanethiol, dibenzothiophene
7	Tert-butyl disulfide, dibenzothiophene
8	Ν-butyl sulfide, benzothiophene, dibenzothiophene
9	1-dodecanethiol, n-butyl sulfide, tert-butyl disulfide, benzothiophene, dibenzothiophene
10	1-dodecanethiol, benzothiophene, dibenzothiophene
11	1-dodecanethiol, n-butyl sulfide, tert-butyl disulfide

**Table 2:** Sulfur compounds, solvent to feed ratio, and sulfur content of the oxidized and extracted products using acetonitrile and methanol.

Sulfur compound	Sulfur content (% w/w) (after oxidation)	Solvent to feed ratio	Acetonitrile		Methanol	
			Yield (% w/w)	Sulfur content (% w/w)	Yield (% w/w)	Sulfur content (% w/w)
1-dodecanethiol	0.340	1.0	94	0.300	99	0.307
		2.0	92	0.276	98	0.288

Ν-butyl sulfide	0.027	1.0	99	0.006	99	0.006
		2.0	94	0.006	97	0.007

Tert-butyl disulfide	0.225	1.0	95	0.152	96	0.156
		2.0	92	0.135	97	0.136

Benzothiophene	0.019	1.0	95	0.005	99	0.006
		1.5	94	0.006	98	0.006
		2.0	92	0.005	97	0.005

Dibenzothiophene	0.005	1.0	99	0.002	99	0.003
		2.0	98	0.001	96	0.002

**Table 3:** IR absorptions of the model sulfur compounds in their initial solutions and during their oxidation. Characteristic groups that contribute to the monitoring of the oxidative process. The absorptions of the solvent, n-hexadecane, were excluded.

Characteristic groups	1-dodecanethiol	N-butyl sulfide	Tert-butyl disulfide	Benzothiophene	Dibenzothiophene
*IR absorptions of the sulfur compounds in their initial solutions (cm * ^*−1*^)					
Tertiary group C-H bending	—	—	1363	—	—
=CH- skeletal vibrations	—	1273, 1220	—	—	—
C-C skeletal vibrations	—	—	1161	—	—
Aromatic C-H in-plane bending	—	—	—	1207, 1157, 1089, 1055, 1014	1227, 1197, 1160, 1075, 1068, 1026
Aromatic C-H out-of-plane bending	—	—	—	880, 866, 848, 798, 760, 734	740

*IR absorptions used to monitor the oxidation of the solutions (cm * ^*−1*^)					
SO_2_ symmetric stretching	1133	1136	—	—	—
S=O stretching vibrations	1060	—	1060	—	—
Aromatic C-H out-of-plane bending	—	—	—	—	740

**Table 4:** Sulfur compounds contained in each mixture, solvent to feed ratio, sulfur content of the oxidized and extracted products, and mass yields using acetonitrile and methanol.

Mixture	Sulfur compounds	Sulfur content (% w/w) (after oxidation)	Solvent to feed ratio	Acetonitrile		Methanol	
				Yield (% w/w)	Sulfur content (% w/w)	Yield (% w/w)	Sulfur content (% w/w)
1	Tert-butyl disulfide, benzothiophene	0.124	1.0	94	0.056	97	0.073
			1.5	94	0.048	97	0.062
			2.0	95	0.042	96	0.056

2	1-dodecanethiol, tert-butyl disulfide	0.267	1.0	93	0.219	98	0.225
			1.5	93	0.209	94	0.216
			2.0	92	0.205	95	0.213

3	1-dodecanethiol, n-butyl sulfide	0.081	1.0	93	0.063	94	0.066
			1.5	88	0.057	93	0.062
			2.0	87	0.053	91	0.057

4	Ν-butyl sulfide, tert-butyl disulfide	0.019	1.0	95	0.010	97	0.009
			1.5	95	0.009	95	0.009
			2.0	92	0.009	95	0.008

5	1-dodecanethiol, benzothiophene	0.108	1.0	90	0.061	92	0.071
			1.5	96	0.054	91	0.063
			2.0	98	0.050	91	0.058

6	1-dodecanethiol, dibenzothiophene	0.085	1.0	97	0.063	96	0.066
			1.5	97	0.059	95	0.062
			2.0	97	0.054	97	0.058

7	Tert-butyl disulfide, dibenzothiophene	0.046	1.0	96	0.027	94	0.031
			1.5	95	0.024	95	0.028
			2.0	92	0.022	93	0.026

8	Ν-butyl sulfide, benzothiophene, dibenzothiophene	0.028	1.0	91	0.006	94	0.006
			1.5	92	0.005	93	0.005
			2.0	89	0.005	94	0.005

9	1-dodecanethiol, n-butyl sulfide, tert-butyl disulfide, benzothiophene, dibenzothiophene	0.069	1.0	92	0.036	95	0.039
			1.5	93	0.032	93	0.039
			2.0	93	0.030	94	0.035

10	1-dodecanethiol, benzothiophene, dibenzothiophene	0.026	1.0	94	0.013	94	0.015
			1.5	92	0.012	94	0.014
			2.0	93	0.011	92	0.013

11	1-dodecanethiol, n-butyl sulfide, tert-butyl disulfide	0.129	1.0	95	0.091	94	0.096
			1.5	94	0.080	94	0.080
			2.0	95	0.071	95	0.087

**Table 5:** IR absorptions of the surrogate fuels in their initial mixtures and during their oxidation. Characteristic groups that contribute to the monitoring of the oxidative process. The absorptions of the solvent, n-hexadecane, were excluded.

Characteristic groups	Mixture 1	Mixture 2	Mixture 3	Mixture 4	Mixture 5	Mixture 6	Mixture 7	Mixture 8	Mixture 9	Mixture 10	Mixture 11
*IR absorptions of the surrogate fuels in their initial mixtures (cm * ^*−1*^ * )*											

Tertiary group C-H bending	—	—	—	—	—	—	—	—	—	—	*—*

=CH- skeletal vibrations	—	—	—	—	—	—	—	—	—	—	*—*

C-C skeletal vibrations	1161	1161	—	1161	—	—	—	—	—	—	*—*

Aromatic C-H in-plane bending	1089, 1055, 1014	—	—	—	1089, 1055, 1014	1227, 1160, 1075, 1068, 1026	1227, 1160, 1075, 1068, 1026	1227, 1160, 1089, 1075, 1068, 1026	1228, 1160, 1089, 1075, 1026	1228, 1160, 1089, 1075, 1056, 1026	*—*

Aromatic C-H out-of-plane bending	866, 798, 760, 734	—	—	—	866, 798, 760, 734	740	740	866, 797, 760, 741	798, 760, 740	866, 848, 798, 760, 740	*—*

*IR absorptions used to monitor the oxidation of the mixtures (cm * ^*−1*^ * )*											

SO_2_ symmetric stretching	—	1133	1135	1133	—	—	—	—	1133	—	1135

S=O stretching vibrations	—	1060	1061	—	—	—	—	—	—	—	1061

Aromatic C-H out-of-plane bending	760	—	—	—	760	740	740	760	*—*	*—*	*—*

## Data Availability

The data used to support the results of this study are included within the article. Any further information is available from the corresponding author upon request.
